# Beyond existing rating scales: development of a novel nomogram for predicting severe clinical bleeding associated with low-molecular-weight heparin in hospitalized medical patients

**DOI:** 10.1007/s11096-025-02070-3

**Published:** 2025-12-15

**Authors:** Zailin Fu, Xia Zhan, Min He, Zhijun Dong, Yuanyuan Fang, Xiaoying Zhang, Ting Zhou, Bo Jin, Dabu Zhu, Jianrong Gu, Yi Zhou, Yifang Chen, Minghua Xie, Hong Yuan

**Affiliations:** 1 Department of Pharmacy, Linping Campus, The Second Affiliated Hospital, Zhejiang University School of Medicine, Hangzhou, 310000 Zhejiang People’s Republic of China; 2 Department of Pharmacy, The First People’s Hospital of Lin’an District, Hangzhou, 310000 Zhejiang People’s Republic of China; 3 Department of Pharmacy, The First People’s Hospital of Xiaoshan District, Hangzhou, 310000 Zhejiang People’s Republic of China; 4 Department of Pharmacy, Hangzhou Normal University Jiande Hospital, Hangzhou, 310000 Zhejiang People’s Republic of China; 5 Department of Cardiovascular Medicine, Linping Campus, The Second Affiliated Hospital, Zhejiang University School of Medicine, #369 Yingbin Road, Linping, Hangzhou, 310000 Zhejiang People’s Republic of China

**Keywords:** Hemorrhage/epidemiology, Low-molecular-weight heparin/adverse effects, Nomograms, Predictive value, Risk assessment, Risk factors

## Abstract

**Introduction:**

Low-molecular-weight heparin (LMWH) is widely used for thromboprophylaxis and treatment in hospitalized patients; however, LMWH-related severe clinical bleeding (LSCB) remains a major concern. Existing risk scales have been developed for oral anticoagulants and have limited applicability to LMWH, leaving clinicians without reliable bedside tool.

**Aim:**

This study aimed to evaluate the discriminatory performance of existing scales for predicting LSCB and develop a tailored nomogram for individualized risk prediction.

**Method:**

Hospitalized medical patients prescribed LMWH between July 2021 and August 2024 at three tertiary hospitals in Hangzhou, China were retrospectively analyzed. Each LSCB case was matched with three non-LSCB controls from the same department and period. The prevalence of LSCBs, bleeding sites, and clinical characteristics are described. Receiver operating characteristic (ROC) curves were used to assess the predictive performance of existing scales. Variables with *p* < 0.10 in univariate analysis were entered into logistic regression, a backward stepwise elimination (stay *p* < 0.05) was applied to identify independent predictors and subsequently incorporated into a nomogram. Discrimination, calibration, and external validation were performed.

**Results:**

Among 22,096 patients, 369 (1.67%) developed LSCB, most commonly severe gastrointestinal bleeding (74.3%), with a mean onset of 5.68 days. A total of 1,089 patients with non-LSCB were matched as controls. Existing scales performed limited predictive value (AUC 0.52–0.68). Logistic regression identified 12 independent predictors: hypoproteinemia (albumin < 30 g/L), anemia (Hb < 90 g/L), active gastrointestinal ulcer, thrombocytopenia (platelets < 75 × 10⁹/L), coagulation abnormalities (PT or aPTT > 1.2 × ULN), cefoperazone/latamoxef exposure > 7 days, hypocalcemia ([Ca^2^⁺] < 2.10 mmol/L), aspirin therapy, dual antiplatelet therapy, renal dysfunction (GFR < 60 mL/min), hepatic impairment (AST or ALT ≥ 3 or TBIL ≥ 2 × ULN), and age > 65 years. Odds ratios ranged from 6.16 (hypoproteinemia) to 1.47 (age > 65 years). A nomogram, named LSCB-Score, incorporating these factors achieved AUC 0.890 in the derivation cohort. Calibration was good (Hosmer–Lemeshow *p* = 0.312), and predictions closely matched the observations. External validation yielded an AUC of 0.876, confirming robustness.

**Conclusion:**

The existing scales for predicting LSCB lack accuracy in hospitalized patients. This newly developed nomogram (LSCB-Score) provides a practical framework for individualized bleeding risk assessment and facilitates safe management of LMWH in hospitals.

**Supplementary Information:**

The online version contains supplementary material available at 10.1007/s11096-025-02070-3.

## Impact statements

- This study highlights that the existing bleeding risk scales are insufficient for hospitalized LMWH users, demonstrating the need for new tools.
- The identification of hypoproteinemia, hypocalcemia, and cefoperazone/latamoxef exposure increases clinicians’ awareness of previously overlooked bleeding risk factors.
- The validated nomogram offers clinicians a practical decision-support tool to personalize anticoagulation therapy and enhance patient safety.

## Introduction

Low-molecular-weight heparin (LMWH) is the most widely used agent for therapeutic and prophylactic anticoagulation in the hospital setting. Data from studies such as ENDORSE indicate that nearly 80% of patients with venous thromboembolism (VTE) or those at elevated risk receive LMWH during hospitalization [1]. In line with this, the China National Health Commission, for several consecutive years, listed “enhancing the standardized prevention rate of VTE” as a national medical quality and safety improvement goal [2].

Although the widespread adoption of LMWH has reduced the incidence of VTE, it has also led to another clinical concern: LMWH-related severe clinical bleeding (LSCB) [3, 4]. LSCB is defined as bleeding at critical sites, any overt bleeding, or any bleeding requiring urgent therapeutic intervention, provided that such events are causally linked to the administration of LMWH. In most cases, clinicians only assess whether hospitalized patients have contraindications to LMWH before initiating the therapy. However, relying solely on contraindications is insufficient to meaningfully reduce the incidence of LSCBs. Even among patients without contraindications, LSCB occurs in 2.0–4.7% of patients with acute coronary syndrome (ACS) and in 1.5–2.1% of those receiving VTE prophylaxis or treatment [5–7]. LSCB are a leading cause of discontinuation of anticoagulation therapy and remain a major limitation of in-hospital anticoagulation. It not only reduces the net clinical benefit of treatment but also prolongs hospital stay and increases mortality risk [8].

To estimate bleeding risk, numerous assessment tools, such as HAS-BLED, ORBIT, ATRIA, HEMORR2HAGES, Shireman, OBRI, ACCP, VTE-BLEED, Hokusai, Seiler, IMPROVE, and RIETE, have been developed in recent years [9, 10]. However, several challenges remain to be overcome. Most of these tools are were derived from cohorts receiving long-term oral anticoagulation: either vitamin-K antagonists or direct oral anticoagulants for stroke prevention in atrial fibrillation or for extended treatment of venous thromboembolism (Supplementary Tables S1) [9, 11–23]. The datasets used for deriving these tools contain a minimal number of patients treated with LMWH. Therefore, their predictive performance for LSCB requires further validation. Additionally, existing tools are primarily intended for patients in stable, long-term anticoagulation phases [24, 25], whereas hospitalized patients often present with more complex conditions such as hypoproteinemia, hypocalcemia, acute hepatic or renal insufficiency, coagulopathy, anemia, and severe infections. Moreover, the concurrent use of medications, such as glucocorticoids, nonsteroidal anti-inflammatory drugs (NSAIDs), P2Y12 receptor antagonists, cefoperazone, and latamoxef, may further increases the risk of bleeding.

These complicating factors limit the applicability of the existing bleeding risk scales, which have been primarily developed for outpatient or community settings. Some predictors included in these tools—such as alcohol abuse and frequent falls—are less relevant for inpatients [11, 14, 26], while others, including “international normalized ratio (INR) > 1.5” or “time-in-therapeutic window (TTR) < 60%,” are specific to warfarin use [26]. As a result, their use in hospitalized populations has important shortcomings.

Given these limitations, it is crucial to critically assess the predictive performance of the available risk assessment tools for LSCB and to develop a dedicated model specifically tailored to hospitalized patients receiving LMWH.

### Aim

This study aimed to evaluate the discriminatory performance of existing bleeding risk scales for predicting LMWH-related severe clinical bleeding in hospitalized patients and develop a tailored nomogram for individualized risk prediction.

## Method

### Study design and participants

This retrospective study extracted clinical data from the electronic medical records of three tertiary hospitals in Hangzhou, China. Medical patients who underwent LMWH (enoxaparin or nadroparin) for venous thromboembolism (VTE) and/or atrial fibrillation (AF) between July 2021 and August 2024 were eligible. Participants were categorized into the LSCB and non-LSCB groups.

In accordance with the definitions provided by the International Society on Thrombosis and Haemostasis (ISTH) for "major bleeding" and "clinically relevant non-major bleeding", the LSCB group included patients with bleeding at critical anatomical sites (such as intracranial, retroperitoneal, intraocular, and pericardial locations), severe gastrointestinal bleeding (e.g., hematemesis, melena), significant urinary bleeding (gross hematuria > 24 h), extensive subcutaneous hemorrhage, excessive menstrual bleeding (> 200 mL/day), or persistent epistaxis.

All exclusion criteria were selected to remove competing causes of bleeding that would obscure a causal link between LMWH exposure and LSCB; accordingly, patients were excluded for any of the following: pre-existing bleeding prior to LMWH initiation; concomitant use of other anticoagulants; known coagulopathies; ongoing dialysis or end-stage renal disease; moderate-to-severe renal insufficiency (glomerular filtration rate [GFR] 15–30 mL/min) without appropriate LMWH dose adjustment; Child–Pugh score ≥ 10; substantial deviations from recommended LMWH dosing; LMWH treatment duration < 24 h; platelet counts < 30 × 10⁹/L; severe clinical bleeding within the previous 30 days; suspected esophageal varices; or arteriovenous malformations/hemangiomas.

The non-LSCB group included patients without LSCB or with only minor bleeding events that did not meet the LSCB criteria. To ensure sufficient statistical power for detecting exposure differences between cases and controls while minimizing over-matching bias, each LSCB case was matched chronologically with three non-LSCB controls admitted to the same department during the corresponding time frame. Patients with LSCB and those with incomplete clinical data were excluded.

## Data collection

A structured data collection template was developed using Microsoft Excel 2010 to capture potential risk factors. Patient information, including demographics, comorbidities, laboratory results, concomitant medications, and prior bleeding history, was extracted from the electronic medical records. After dual review and verification, the data were entered into the database and duplicate patient records were removed.

Collected variables included demographic factors (age, sex, and weight) and laboratory values (platelet count, hemoglobin [Hb], creatinine, alanine aminotransferase [ALT], aspartate aminotransferase [AST], total bilirubin [TBIL], plasma albumin, plasma calcium, prothrombin time [PT], activated partial thromboplastin time [aPTT], and INR). Comorbidities recorded included diabetes mellitus, uncontrolled hypertension, heart failure, respiratory failure, malignancy, active gastrointestinal ulcer, history of stroke, severe infection and atherosclerotic cardiovascular disease. All comorbidities previously encompassed by above 12 scales were incorporated into the comorbidity spectrum of this study. Concomitant medication use, including glucocorticoids, aspirin, P2Y12 receptor antagonists, cefoperazone, and latamoxef was also noted.

Quantitative data were prioritized as follows: values within 24 h before LSCB onset, followed by values within 24–48 h before LSCB, and within 24 h after LSCB. For non-LSCB patients, laboratory values obtained during days 2–8 of LMWH therapy were averaged, as most LSCB events occurred within this timeframe. The glomerular filtration rate (GFR) was calculated using the Cockcroft-Gault equation.

As the study population comprised hospitalized patients, certain clinical data with low incidence, unavailability, or difficulty in monitoring (e.g., frequent falls, alcohol abuse, genetic predisposition, and low physical activity) were coded as “none” and assigned a value of “0”. Risk scores for LSCB were calculated according to the classifications defined in the original validation of each bleeding risk scale [27].

## Statistical analysis

All analyses were performed using SPSS version 26.0 and R version 4.4.2. Categorical variables are expressed as frequencies and percentages, and continuous variables as mean ± standard deviation (SD). Group comparisons were conducted using the chi-square test or Fisher’s exact test for categorical variables and Student’s t-test for continuous variables.

The predictive performance of each bleeding risk scale for in-hospital LSCB was evaluated by calculating the area under the receiver operating characteristic (ROC) curve (AUC). Univariate analysis was first conducted, followed by binary logistic regression, to identify independent risk factors for LSCB. Odds ratios (ORs) with 95% confidence intervals (CIs) were reported to indicate the magnitude of the risk. Variables with p < 0.05 were incorporated into the final model.

A nomogram was constructed based on the identified risk factors. Model performance was assessed using ROC curves for discrimination, and calibration was evaluated using calibration plots and the Hosmer–Lemeshow goodness-of-fit test. The discrimination was classified as poor (AUC 0.51–0.69), acceptable (0.70–0.79), excellent (0.80–0.89), or outstanding (0.90–1.00) [28].

### Ethics approval

This study was approved by the Ethics Committee of the Linping Campus, the Second Affiliated Hospital, Zhejiang University School of Medicine (approval number: 2024-02-027; approved 22 Feb 2024). 

## Results

### Epidemiology

A total of 22,096 hospitalized patients underwent LMWH. Among them, 369 (1.67%) met the diagnostic criteria for LSCB. Linping Hospital reported 137 cases, Lin’an Hospital 119 cases, and Xiaoshan Hospital 113 cases. Six patients were excluded from the ROC and regression analyses due to incomplete data but were included in the descriptive analyses. A total of 1,089 patients with non-LSCB were chronologically matched as controls.

**Fig. 1:**
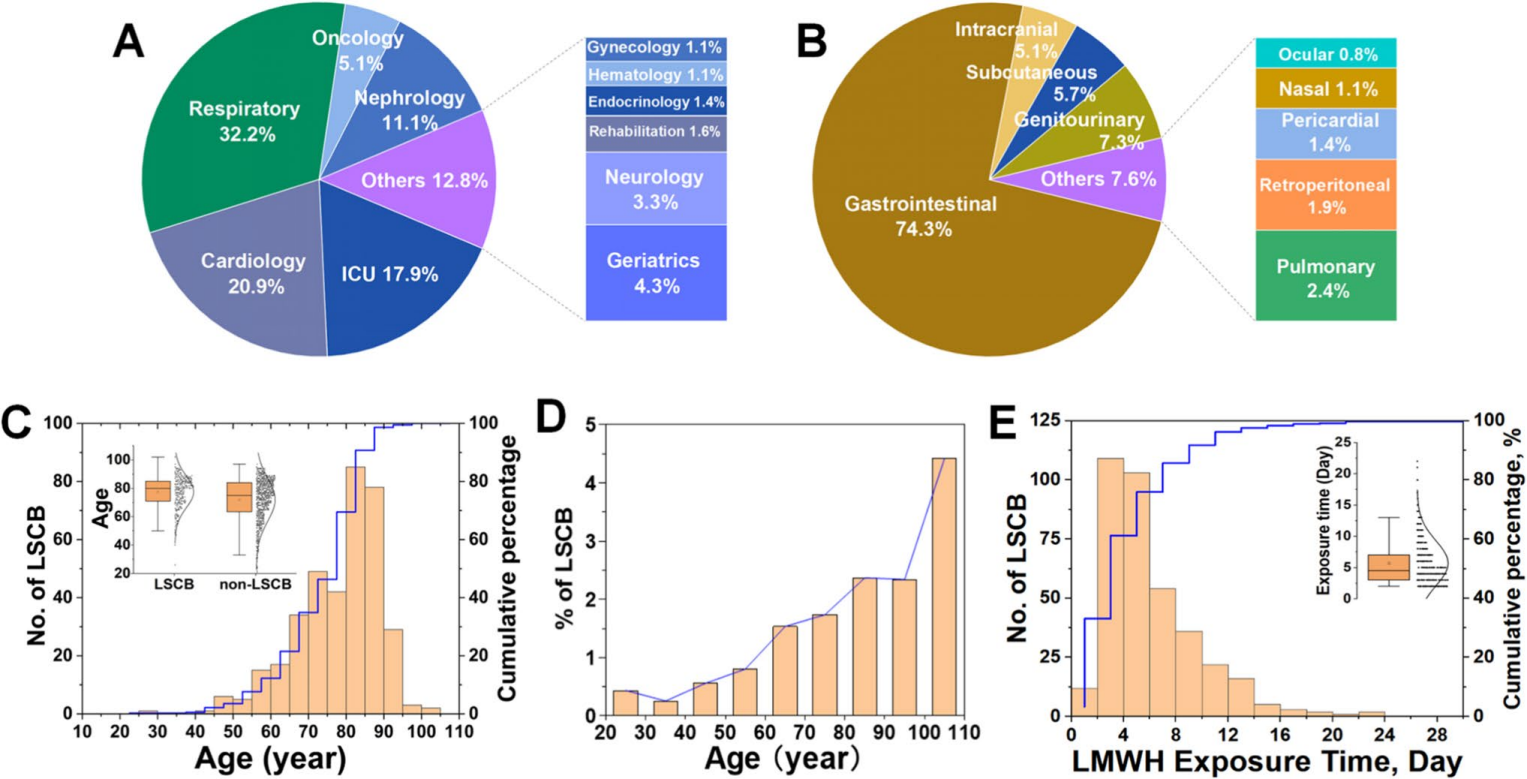
Epidemiology of LMWH-related severe clinical bleeding (LSCB) in hospitalized medical patients. **A** Departmental Distribution of LSCB Cases. (B) Primary bleeding sites. **C** Age distribution of patients with LSCB. **D** Incidence of LSCB across age groups. **E** Time to onset of LSCB events

Most LSCB cases were reported in respiratory medicine (32.2%), cardiology (20.9%), intensive care (17.9%), nephrology (11.1%), and oncology (5.1%) (Fig. 1A). Among the primary source departments for LSCB cases, cardiology exhibited a slightly higher LSCB rate (2.14%), than those of respiratory medicine (1.87%) and ICU (2.04%). However, χ^2^ testing confirmed no statistically significant difference among the three. Severe gastrointestinal bleeding was the most frequent (74.3%), followed by genitourinary bleeding, subcutaneous hemorrhage, and intracranial hemorrhage **(**Fig. 1B). Approximately 90% of patients with LSCB were aged ≥ 60 years, with a prevalence ranging from 0.3–0.8% in younger adults to 2–5% in the elderly (Fig. 1C, 1D). Most events occurred between days 2 and 8 of the LMWH therapy, with a mean onset of 5.68 days (Fig. 1E).

## Baseline characteristics

The baseline characteristics of patients with and without LSCB are summarized in Table 1. No significant differences were observed in the body mass index (BMI) or LMWH dosing regimens (p > 0.05). Univariate analyses showed no significant group differences in dyslipidemia, uncontrolled hypertension, respiratory failure, diabetes mellitus, heart failure or severe infection.

**Table 1:** Baseline characteristics of enrolled medical patients

Characteristics	Participants, n (%)			*P* value
	Total (*n* = 1,452)	LSCB (*n* = 363)	Non-LSCB (*n* = 1,089)	
*Patient Demographics*				
Age, Mean (SD), years	73.2 (14.3)	77.4 (11.5)	71.2 (15.1)	< 0.001
> 65	1,125 (77.5)	320 (88.2)	805 (73.9)	< 0.001
> 80	589 (40.6)	201 (55.4)	388 (35.6)	< 0.001
*Sex*				
Male	789 (54.3)	201 (55.4)	588 (54.0)	0.65
Female	663 (45.7)	162 (44.6)	501 (46.0)	
Body Weight, Mean (SD), kg	61.2 (11.2)	60.2 (10.9)	61.5 (11.4)	0.06
BMI, Mean (SD), kg/m^2^	22.9 (3.7)	22.6 (3.8)	23.0 (3.7)	0.08
*LMWH Dosage Regimen*				
Prophylactic Dose	1,148 (79.1)	277 (76.3)	871 (80.0)	0.14
Therapeutic Dose	304 (20.9)	86 (23.7)	218 (20.0)	
LMWH Preparations				
Enoxaparin	1,197 (82.4)	304 (83.7)	893 (82.0)	0.47
Nadroparin	255 (17.6)	59 (16.3)	196 (18.0)	
*COMORBIDITIES*				
Diabetes Mellitus	392 (27.0)	111 (30.6)	281 (25.8)	0.09
Malignant Tumor	197 (13.6)	65 (17.9)	129 (11.8)	< 0.001
Dyslipidemia	140 (9.6)	38 (10.5)	102 (9.4)	0.54
Uncontrolled Hypertension^a^	115 (7.9)	24 (6.6)	95 (8.7)	0.23
Respiratory Failure	418 (28.8)	117 (32.2)	301 (27.6)	0.10
Heart Failure	255 (17.6)	76 (20.9)	179 (16.4)	0.06
History of stroke	148 (10.2)	39 (10.7)	109 (10.0)	0.69
Active Gastrointestinal Ulcer	82 (5.6)	41 (11.3)	40 (3.7)	< 0.001
Severe Infection^b^	119 (8.2)	33 (9.1)	86 (7.9)	0.51
ASCVD	399 (27.5)	114 (31.4)	285 (26.2)	0.06
*Laboratory Tests*				
Thrombocytopenia				
Pt Count, Mean (SD), × 10^9^/L	194 (94)	189 (102)	196 (91)	0.17
Pt Count < 100 × 10^9^/L	267 (18.4)	77 (21.2)	190 (17.4)	0.11
Pt Count < 75 × 10^9^/L	96 (6.6)	44 (12.1)	52 (4.8)	< 0.001
*Anemia*				
Hb, Mean (SD), g/L	104.5 (24.3)	92.5 (23.2)	111.8 (22.0)	< 0.001
Hb < 130 g/L ♂ or < 120 g/L ♀	1103 (76.0)	301 (82.9)	802 (73.6)	< 0.001
Hb < 90 g/L	339 (23.3)	178 (49.0)	161 (14.8)	< 0.001
*Hypoproteinemia*				
Albumin, Mean (SD), g/L	32.6 (5.0)	28.9 (4.2)	33.8 (4.5)	< 0.001
Albumin < 30 g/L	439 (30.2)	226 (62.3)	213 (19.6)	< 0.001
*Hepatic Impairment*				
AST and/or ALT ≥ 3 ULN and/or TBIL ≥ 2 ULN	187 (12.9)	71 (19.6)	116 (10.7)	< 0.001
*Renal Dysfunction*				
GFR < 60 ML/MIN	591 (40.7)	197 (54.3)	394 (36.2)	< 0.001
Clotting abnormalities				
PT and/or aPTT > 1.2ULN	310 (21.3)	148 (40.8)	162 (14.9)	< 0.001
INR > 1.5	93 (6.4)	35 (9.6)	58 (5.3)	0.002
*Hypocalcemia*				
[Ca^2+^], Mean (SD), mmol/L	2.14 (0.19)	2.06 (0.15)	2.17 (0.19)	< 0.001
[Ca^2+^] < 2.10 mmol/L	588 (40.5)	222 (61.2)	366 (33.6)	< 0.001
*S*				
Corticosteroids	314 (21.6)	83 (22.9)	213 (19.6)	0.18
Aspirin	325 (22.4)	99 (27.3)	226 (20.8)	0.01
Cilostazol or Indobufen	88 (6.1)	27 (6.9)	61 (5.6)	0.37
P2Y12 Receptor Antagonists	232 (16.0)	65 (17.9)	167 (15.3)	0.25
Dual Antiplatelet^c^	104 (7.2)	35 (9.6)	69 (6.3)	0.05
Cefperazone or Latamoxef	329 (22.7)	112 (30.8)	217 (19.9)	< 0.001
PPIs	582 (40.1)	171 (47.1)	461 (42.3)	0.11

Qualitative variables are presented as n (%) and quantitative variables are presented as mean (SD)

^a^Systolic blood pressure > 160 mmHg within the 48 h prior to bleeding

^b^Adhere to the criteria for systemic inflammatory response and possess a SOFA ≥ 2

^c^Aspirin plus another antiplatelet drug

LSCB, LMWH-related severe clinical bleeding; BMI, body mass index; ASCVD, atherosclerotic cardiovascular disease; Pt, platelet count; Hb, hemoglobin; AST, aspartate transaminase; ALT, alanine transaminase; TBIL, total bilirubin; ULN, upper limit of normal value; GFR, Glomerular filtration rate; APTT, activated partial thromboplastin time; PT, prothrombin time; INR, international normalized ratio; [Ca^2+^], plasma calcium concentration; PPIs, proton pump inhibitors

Combination therapy with P2Y12 receptor antagonists, cilostazol, indobufen, or glucocorticoids showed no significant association with LSCB. In contrast, co-administration of aspirin or dual antiplatelet therapy (DAPT, aspirin plus another antiplatelet agent) was significantly associated with LSCB. Patients with LSCB also had a higher prevalence of older age, malignancy, active gastrointestinal ulcers, thrombocytopenia, anemia, hypoproteinemia, hepatic impairment, coagulation abnormalities, renal dysfunction, hypocalcemia, and cefoperazone/latamoxef exposure.

The proportion of patients receiving therapeutic LMWH doses was not significantly higher in the LSCB group than in the control group (*p* = 0.14). However, subgroup analysis showed that among patients with hypoproteinemia, therapeutic dosing was associated with a higher risk of LSCB than prophylactic dosing (Table 2).

**Table 2:** LSCB risk in hypoproteinemic patients exposed to prophylactic or therapeutic Doses of LMWH^a^

Characteristics	Prophylactic Dose	Therapeutic Dose	*P* value
	(*n* = 3,769)	(*n* = 994)	
Age, Mean (SD), years	72.2 (13.2)	72.9 (12.4)	0.11
Active Malignant Tumor	551 (14.6)	122 (12.3)	0.065
Active Gastrointestinal Ulcer	383 (10.2)	84 (8.5)	0.12
Hepatic Impairment	547 (12.9)	156 (16.5)	0.37
Renal Dysfunction	1,196 (33.3)	284 (28.6)	0.059
Antiplatelet Therapy	1,217 (26.1)	306 (20.9)	0.38
LSCB	158 (4.2)	68 (6.8)	< 0.001
Severe Gastrointestinal Hemorrhage	128 (3.4)	47 (4.7)	< 0.001
Other LSCB	30 (0.8)	21 (2.1)	< 0.001

^a^Among 22,096 medical patients, 4,763 had hypoproteinemia. Hypoproteinemia was defined as a mean albumin level < 30 g/L within days 2–8 of LMWH therapy in non-LSCB patients or an albumin level < 30 g/L prior to LSCB onset in LSCB patients

## Risk categorization by existing scales

**Fig. 2:**
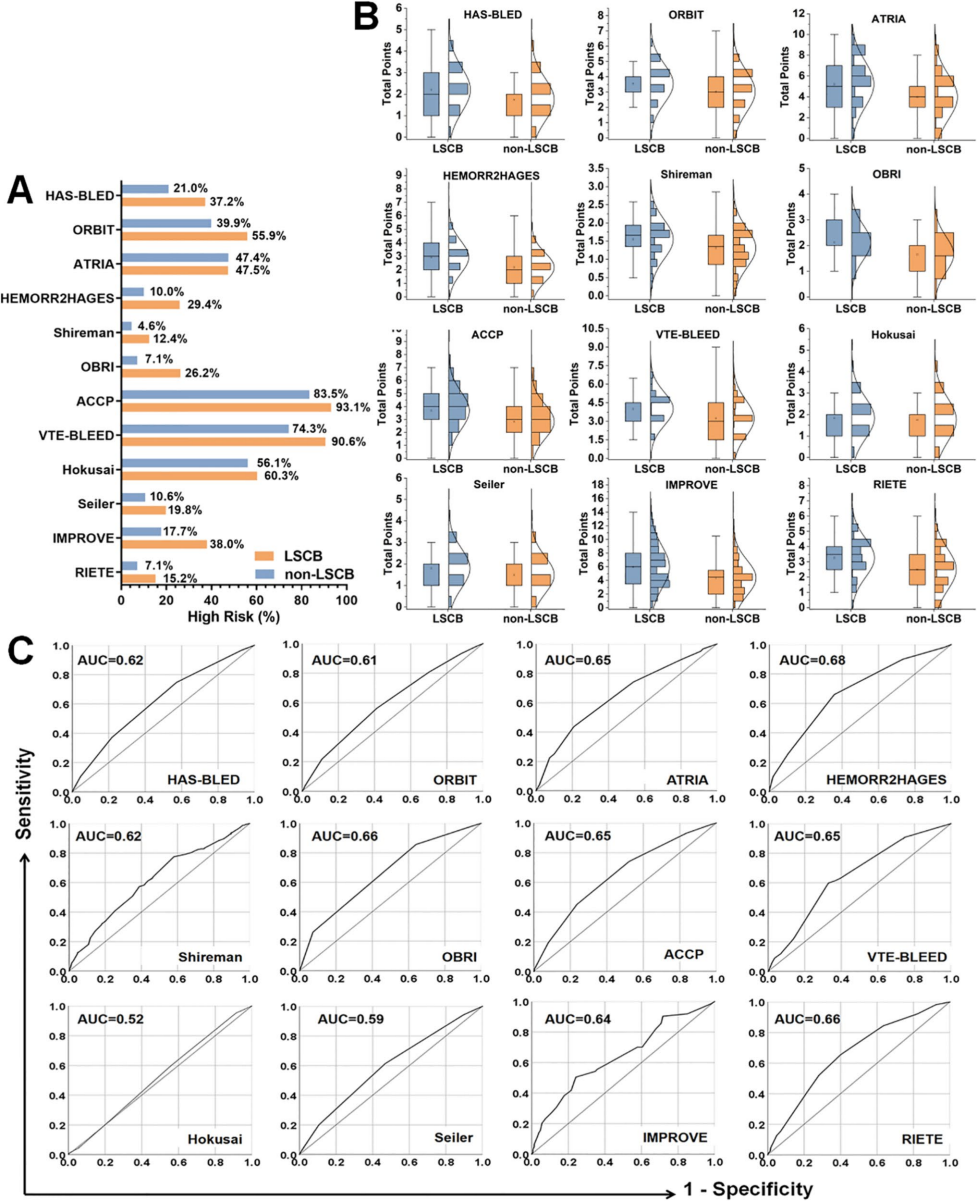
Performance of existing bleeding risk scales for predicting LMWH-related severe clinical bleeding (LSCB). **A** Proportion of patients classified as high risk in LSCB and non-LSCB groups. **B** Score distributions across scales. **C** Receiver operating characteristic (ROC) curves of the scales with corresponding area under the curve (AUC) values

Risk stratification results based on the existing bleeding risk scores are presented in Supplementary Tables S2-S3 and Fig. 2A. Except for VTE-BLEED and IMPROVE, which used binary cutoffs, most scales categorized patients into low-, medium-, and high-risk groups. Shireman et al. underestimated the proportion of high-risk patients in their LSCB group (12.4%, 15.2%, and 19.8%, respectively). Conversely, Hokusai, VTE-BLEED, and ACCP classified large proportions of non-LSCB patients as high-risk (56.1%, 74.3%, and 83.5%, respectively). Score distributions were generally similar between the groups, except for HEMORR2HAGES, OBRI, and RIETE, which showed slightly greater differences (Fig. 2B).

## Predictive performance of existing scales

ROC analyses showed limited predictive performance of the existing bleeding risk scales, with AUCs ranging from 0.52 to 0.68 (Fig. 2C). The sensitivity and specificity values based on dichotomized categories are provided in Supplementary Table S4. Shireman, OBRI, HEMORR2HAGES, Seiler, and IMPROVE showed high specificity (86.4–95.4%) but low sensitivity (12.5–38.3%). ACCP and VTE-BLEED showed high sensitivity (90.7–93.2%) but low specificity (16.4–25.7%). Other scales demonstrated moderate sensitivity (37.1–69.8%) and specificity (43.8–79.0%).

## Independent predictors of LSCB

**Fig. 3:**
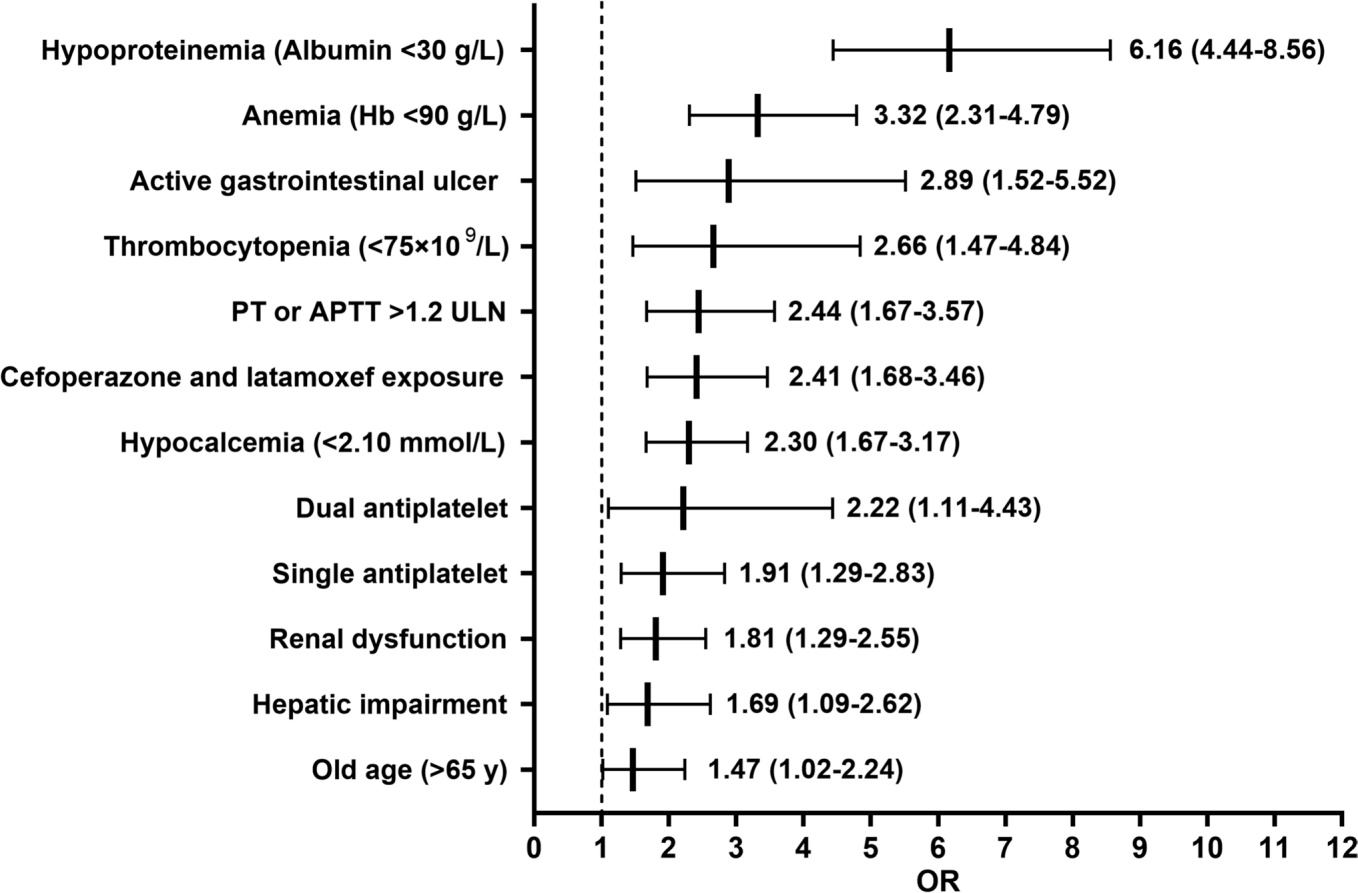
Independent risk factors for LMWH-related severe clinical bleeding (LSCB) identified by binary logistic regression analysis

We first conducted univariable analyses for all candidate variables. Variables with p < 0.10 in univariable analyses were then entered into a binary logistic regression analysis. Subsequently, a backward stepwise elimination procedure (with p < 0.05 as the retention criterion) was applied to identify the final set of independent predictors. Twelve independent predictors of LSCB were identified: hypoproteinemia (albumin < 30 g/L), anemia (Hb < 90 g/L), active gastrointestinal ulcer, thrombocytopenia (platelets < 75 × 10⁹/L), coagulation abnormalities (PT or APTT > 1.2 × ULN), cefoperazone/latamoxef exposure > 7 days, hypocalcemia ([Ca^2^⁺] < 2.10 mmol/L), DAPT, aspirin therapy, renal dysfunction (GFR < 60 mL/min), hepatic impairment (AST or ALT ≥ 3 × ULN or TBIL ≥ 2 × ULN), and age > 65 years. ORs ranged from 6.16 (hypoproteinemia) to 1.47 (age > 65 years) (Fig. 3). The multicollinearity diagnostics show that the variance inflation factors (VIFs) for all twelve factors range from 1.02 to 1.27, indicating virtually no linear correlation among the independent variables; consequently, the standard errors of the regression coefficients will not be inflated by collinearity (Supplementary Table S5). The number of events per variable (EPV) is 30.2, which significantly exceeds the commonly recommended EPV threshold of 10–20. This indicates a robust statistical power and a minimal risk of overfitting.

The findings of this study further substantiate that the inclusion criteria for certain factors in the existing scales may be excessively permissive. When anemia was defined at the traditional threshold of Hb < 130 g/L for men and < 120 g/L for women, no significant association with LSCB was observed. In contrast, redefining anemia as Hb < 90 g/L markedly improved discrimination (Supplementary Fig. S1). Similarly, the substitution of the oral anticoagulant-specific variable “TTR < 60%” with PT > 1.2 × ULN or aPTT > 1.2 × ULN improved predictive accuracy. Supplementary Fig. S2 shows that this change increased the AUCs from 0.62–0.64 to 0.67–0.69. For thrombocytopenia, the conventional cutoff of < 100 × 10⁹/L did not differentiate LSCB from non-LSCB patients (p = 0.11). In contrast, a lower threshold of < 75 × 10⁹/L was strongly associated with LSCB (OR = 2.66), as demonstrated in Supplementary Fig. S3. In subgroup analyses, several predictors varied according to the bleeding site. For instance, systolic blood pressure > 160 mmHg was significantly associated with intracranial hemorrhage but not with gastrointestinal bleeding (Supplementary Fig. S4).

OR, odds ratio; APTT, activated partial thromboplastin time; PT, prothrombin time; ULN, Upper limit of normal value; Hb, Hemoglobin.

## Nomogram construction and validation

**Fig. 4:**
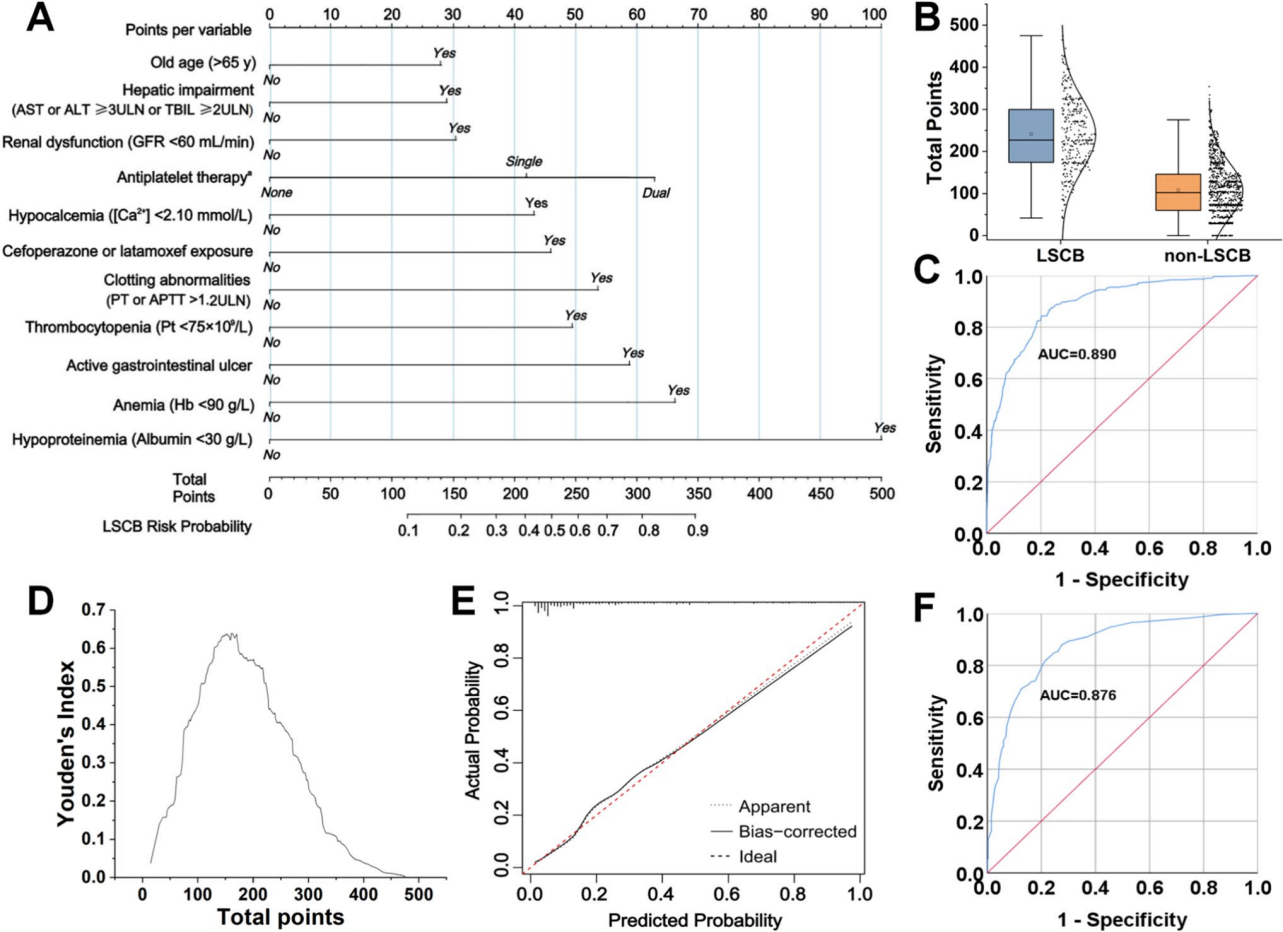
LSCB-Score for predicting LMWH-related severe clinical bleeding and its validation. **A** Point assignment for each variable. **B** Distribution of total scores in LSCB and non-LSCB groups. **C** Receiver operating characteristic (ROC) curve of LSCB-Score. **D** Optimal cut-off score based on the Youden index. **E** Calibration plot. **F** ROC curve in the external validation cohort. ^a^ Single antiplatelet: exposure to aspirin alone; dual antiplatelet: aspirin plus another antiplatelet drug co-exposure. AST, aspartate transaminase; ALT, alanine transaminase; TBIL, total bilirubin; ULN, upper limit of normal value; GFR, Glomerular filtration rate; [Ca^2+^], plasma calcium concentration; APTT, activated partial thromboplastin time; PT, prothrombin time; Pt, Platelet count; Hb, hemoglobin; LSCB, LMWH-related severe clinical bleeding

To improve the practicality and applicability of the logistic regression model, we further transformed this model into a visual nomogram named LSCB-Score (Fig. 4A). According to the nomograph scoring rule, the points for each variable were determined by linearly scaling its regression coefficient to a 0–100 scale. The factor associated with the highest risk is assigned a score of 100 points, while the scores for the remaining variables decrease proportionally. The points assigned were as follows: age > 65 years (28), hepatic impairment (29), renal dysfunction (30), aspirin therapy (42), DAPT (63), hypocalcemia (43), cefoperazone/latamoxef exposure > 7 days (46), coagulation abnormalities (53), thrombocytopenia (50), active gastrointestinal ulcer (59), anemia (67), and hypoproteinemia (100).

Scores were calculated for 1,452 patients. The mean score in the LSCB group was 240.8 versus 107.4 in the non-LSCB group (Fig. 4B). LSCB-Score achieved an AUC of 0.890 (95% CI, 0.870–0.911), exceeding the predictive performance of existing scales (Fig. 4C). Based on the maximum Youden index, the optimal cutoff score was 160, distinguishing high- from low-risk patients (Fig. 4D).

The calibration of LSCB-Score was satisfactory (Hosmer–Lemeshow p = 0.312), and the calibration plot derived from 1000 bootstrap resamples showed close agreement between the predicted and observed outcomes (Fig. 4E). External validation in 138 patients at Linping District First People’s Hospital (23 LSCB events) yielded an AUC of 0.876 (95% CI, 0.849–0.900), confirming the robustness of LSCB-Score (Fig. 4F).

## Discussion

### Key findings

The increasing emphasis on preventing VTE and atrial fibrillation-associated stroke has led to the widespread use of LMWH during hospitalization [29]. Although its efficacy and safety are well established, LSCB remains a major concern, particularly in hospitalized patients. In this study, 1.67% of inpatients treated with LMWH developed LSCB, demonstrating the need for robust risk stratification strategies. Existing bleeding risk scales, largely derived from oral anticoagulant populations, fail to capture the unique pharmacological and physiological contexts of short-term LMWH therapy and therefore do not reliably identify high-risk individuals [30–32].

### Limitations of existing bleeding risk scales

Most available scales showed poor discriminative performance in our cohort (AUC 0.52–0.68), below the clinically useful threshold of ≥ 0.70 [28, 33]. In contrast, the nomogram (LSCB-Score) developed in this study achieved a c-statistic of 0.890, corresponding to a 0.21–0.37 absolute improvement in AUC and the approximate net re-classification improvement (NRI) of 0.30, indicating that roughly one in three patients would be re-classified into a more appropriate risk category. Calibration plots confirmed that predicted vs. observed event rates remained close to the 45° line across the entire risk spectrum. Collectively, these data underscore the incremental value of incorporating hospital-specific predictors and refined laboratory thresholds into a dedicated LMWH-bleeding risk tool.

Several factors contribute to underperformance. First, the anemia thresholds widely used in current scales (Hb < 130 g/L in men and < 120 g/L in women) did not differentiate between the LSCB and non-LSCB groups in our population [34, 35]. In contrast, redefining anemia as Hb < 90 g/L improved discrimination and substantially increased the odds of LSCB, highlighting the importance of applying stricter thresholds for hospitalized patients. Second, the variable “TTR < 60%,” validated for long-term warfarin therapy, is unsuitable for short-course LMWH [11, 20, 36]. Replacing it with PT > 1.2 × ULN or aPTT > 1.2 × ULN improved the model performance and was confirmed by regression as a strong independent predictor (OR 2.44). Although LMWH usually causes only modest PT/aPTT prolongation, elevations above 1.2 × ULN may indicate overexposure to LMWH. In countries where anti-Xa monitoring is not routinely available, PT and aPTT may serve as practical surrogates for detecting excess anticoagulation [37]. Third, the inconsistent definition of thrombocytopenia reduces the predictive value. A platelet threshold of < 100 × 10⁹/L was not associated with LSCB in this cohort study. In contrast, < 75 × 10⁹/L was significantly associated with bleeding, although its effect on overall model performance was modest [14, 20].

### Novel risk factors

This study also identified three previously overlooked predictors: hypoproteinemia, hypocalcemia, and cefoperazone/latamoxef exposure. Patients with albumin levels < 30 g/L showed a markedly higher risk of LSCB (OR 6.16), consistent with prior evidence linking hypoalbuminemia to increased bleeding. The patients with plasma albumin levels < 30 g/L and < 20 g/L exhibit incident rates of anticoagulant-related major bleeding that are 4.5- and 13.1-fold higher, respectively, than those with normal albumin levels [38, 39]. This association is further supported by two recent cohort studies: one identified hypoalbuminemia as a potential risk factor for postoperative gastrointestinal bleeding [40], while the other demonstrated that correcting hypoalbuminemia reduces the risk of anticoagulant-associated gastrointestinal bleeding [41]. In patients with hypoproteinemia, therapeutic LMWH doses were more likely to precipitate LSCB, emphasizing the need for individualized dosing in malnourished or critically ill patients.

Hypocalcemia was also an independent predictor (OR 2.30), likely reflecting impaired activity of calcium-dependent coagulation pathways and platelet function [42, 43]. An association has been described between hypocalcaemia and both the severity of acute non-variceal upper gastrointestinal bleeding and the need for advanced interventions [44]. The prophylactic correction of hypocalcemia is crucial in managing bleeding risk [45].

Finally, prolonged exposure to cefoperazone or latamoxef was strongly associated with LSCB (OR 2.41), exceeding the odds ratios observed for corticosteroids, P2Y12 antagonists, and even aspirin. This aligns with previous reports of cephalosporin-induced hypoprothrombinemia [46, 47] and reflects the local prescribing patterns in China. Therefore, antibiotic selection in patients receiving LMWH should be made cautiously.

### Clinical implications

LSCB-Score developed in this study incorporated both refined thresholds and novel predictors, achieving excellent discrimination (AUC 0.890 in the derivation cohort; 0.876 in the validation cohort) and good calibration. This tool provides clinicians with a practical framework to identify patients at an elevated bleeding risk. By intervening on high-risk factors and adjusting the anticoagulation regimen, it is feasible to maintain effective anticoagulation while concurrently minimizing the LSCB risk.

To bridge the gap between derivation and bedside implementation, we recommend embedding LSCB-Score into the hospital’s clinical-pharmacist workstation or the electronic medical record systems. Of course, developing a web-based or app-based calculator to support practical use is an excellent approach to rapidly expand the clinical application of this nomogram.

### Limitations

This study has several limitations. The study was conducted in three hospitals in Hangzhou, limiting generalizability, especially given the regional use of cefoperazone and latamoxef. Patients who underwent surgery and dialysis were excluded; therefore, the results may not apply to these groups. Risk factors also varied by bleeding site; for example, systolic blood pressure > 160 mmHg strongly predicted intracranial hemorrhage but not gastrointestinal bleeding; however, the model did not differentiate by bleeding type. Patients with absolute contraindications to LMWH were excluded, although in clinical practice, some (e.g., those with acute pulmonary embolism) still require treatment. Finally, LMWH dose adjustments for hepatic or renal dysfunction were not consistently performed, potentially leading to an underestimation of the bleeding risk.

## Conclusion

This study developed and validated a nomogram (LSCB-Score) that integrates refined risk thresholds and novel hospital-specific predictors to assess the risk of LMWH-related severe clinical bleeding in medically hospitalized patients. The model demonstrated superior discrimination and calibration compared to existing bleeding risk scales, providing a clinically applicable tool to support individualized anticoagulation management. By addressing the limitations of the current risk stratification, LSCB-Score offers a step toward safer and more personalized use of LMWH in real-world hospital settings.

## Supplementary Information

Below is the link to the electronic supplementary material.

Supplementary file1 (DOCX 6759 KB)

## Data Availability

The datasets generated and/or analyzed during the current study are available from the corresponding author upon reasonable request. We encourage the use of LSCB-Score for academic and non-commercial purposes free of charge; users are simply required to cite the original paper.
