# Supplementary material for: Beyond existing rating scales: development of a novel nomogram for predicting severe clinical bleeding associated with low-molecular-weight heparin in hospitalized medical patients
Source: Int J Clin Pharm. 2025 Dec 15;48(3):764–76. doi: 10.1007/s11096-025-02070-3 (PMC13176133; doi:10.1007/s11096-025-02070-3)
Supplement: Supplementary file 1 — Supplementary file1 (DOCX 6759 KB) [file 11096_2025_2070_MOESM1_ESM.docx]

**Beyond Existing Rating Scales: Development of a Novel Nomogram for Predicting Severe Clinical Bleeding Associated with Low-Molecular-Weight Heparin in Hospitalized Medical Patients**

Zailin Fu^1^, Xia Zhan^2^, Min He^3^, Zhijun Dong^4^, Yuanyuan Fang^3^, Xiaoying Zhang^3^, Ting Zhou^2^, Bo Jin^1^, Dabu Zhu^1^, Jianrong Gu^1^, Yi Zhou^1^, Yifang Chen^1^, Minghua Xie^1^, Yuan Hong^5^

**Table S1: Indicative uses of existing bleeding risk scales**

| **Developers** | **Scales** | **Indicative Uses** |
| --- | --- | --- |
| **Atrial fibrillation** | | |
| Pisters et al, 2010 | HAS-BLED | VKA-related major bleeding |
| O'Brien et al, 2015 | ORBIT | VKA/dabigatran-related major bleeding |
| Fang et al, 2015 | ATRIA | VKA-related major bleeding |
| Gage et al, 2006 | HEMORR2HAGES | VKA-related major bleeding |
| Shireman et al, 2006 | Shireman | VKA-related bleeding in the elderly |
| Beyth et al, 1998 | OBRI | VKA-related major bleeding |
| **Venous thromboembolism** | | |
| Kearon et al, 2016 | ACCP | VKA/NOACs-related bleeding |
| Klok et al, 2016 | VTE-BLEED | VKA/dabigatran-related bleeding |
| Di Nisio et al, 2017 | Hokusai | VKA/NOACs-related clinically relevant bleeding |
| Seiler et al, 2017 | Seiler | VKA-related bleeding in the elderly |
| Decousus et al, 2011 | IMPROVE | UFH/LMWH-related bleeding |
| Ruíz-Giménez et al, 2008 | RIETE | VKA/LMWH-related bleeding |

Abbreviations: VKA,Vitamin K antagonists; NOACs, Novel oral anticoagulants; UFH, Unfractionated heparin; LMWH, Low molecular weight heparin.

**Table S2: Risk factor scoring criteria and risk stratification**

| **Risk Scores** | **Applicable Risk Factors (score for each factor)** | **Inapplicable Risk Factors**^a^  **(assign as "0")** | **Risk categories** | **Threshold value** |
| --- | --- | --- | --- | --- |
| HAS-BLED | SBP >160 mmHg (1); Dialysis, renal transplant, or serum reatinine >200 µmol/L (1); Cirrhosis, Bilirubin >2 ULN, AST/ALT/ALP >3 ULN (1); Previous stroke (1); Previous major bleed or bleeding predisposition (anemia and/or severe thrombocytopenia) (1); Age >65 (1); APT/NSAIDs (1) | Labile INR;  Alcohol excess | Low | 0-1 |
|  |  |  | Intermediate | 2 |
|  |  |  | High | ≥3 |
| ORBIT | Age ≥75 (1); Hb <130 g/L in men or <120 g/L in women (2); Any previous GI, intracranial or hemorrhagic stroke (2); eGFR <60 mg/dL/1.73 m^2^ (1); APT (1) | NA | Low | 0-2 |
|  |  |  | Intermediate | 3 |
|  |  |  | High | ≥4 |
| ATRIA | Hb <130 g/L in men or <120 g/L in women (3); eGFR < 30 mL/min or dialysis dependent (3); Age >75 (2); Any previous GI, intracranial or hemorrhagic stroke (1); Hypertension (1) | NA | Low | 0-3 |
|  |  |  | Intermediate | 4 |
|  |  |  | High | 5-10 |
| HEMORR2HAGES | End-stage renal disease, end-stage liver disease or cirrhosis (1); Malignancy (1); Age > 75 (1); Aspirin use or thrombocytopenia (1); Any previous GI bleeding, intracranial or hemorrhagic stroke (2); SBP >160 mmHg (1); Hb <130 g/L in men or <120 g/L in women (1); Stroke (1) | Ethanol abuse;  Genetic factors;  Falls risk | Low | 0-1 |
|  |  |  | Intermediate | 2-3 |
|  |  |  | High | ≥4 |
| Shireman | Age ≥70 (0.49); female (0.31); Any previous GI, intracranial or hemorrhagic stroke within 90 days (0.62) or beyond 90 days (0.58); Diabetes (0.27); Anemia (hematocrit of ≤30%) (0.86); APT (0.32) | Alcohol/drug abuse | Low | ≤1.07 |
|  |  |  | Intermediate | 1.08-2.18 |
|  |  |  | High | ≥2.19 |
| OBRI | Age ≥ 65 (1); Previous stroke (1); Previous GI bleed (1); Comorbidity: Myocardial infarction, Creatinine >1.5 mg/dL, Severe anemia (hematocrit<0.30) or Diabetes mellitus (1) | NA | Low | 0 |
|  |  |  | Intermediate | 1-2 |
|  |  |  | High | ≥3-4 |
| ACCP | Age 66–75 (1), >75 (1); Any previous GI, intracranial or hemorrhagic stroke (1); Active cancer (1); Metastatic cancer (1); Renal failure (eGFR <30 mL/min) (1), Liver failure (1); Thrombocytopenia (<100×10^9^/L) (1); Previous stroke (1); Diabetes mellitus (1); Anemia (1); APT (1); Comorbidity: Myocardial infarction, Congestive heart failure, etc (1); NSAIDs (1); Recent surgery (<3 months) (1) | TTR <60%;  Frequent falls;  Alcohol abuse | Low | 0 |
|  |  |  | Intermediate | 1 |
|  |  |  | High | ≥2 |
| VTE-BLEED | Active cancer (2); Male with SBP >140 mmHg (1); Anemia (1.5); Previous Bleeding (1.5); Age ≥60 (1.5); Creatinine clearance <60 mL/min (1.5); | NA | Low | <2 |
|  |  |  | High | ≥2 |
| Hokusai | Female sex (1); APT (1); Hb <100 g/L (1); History of hypertension (1); SBP >160 mmHg (1) | NA | Low | 0 |
|  |  |  | Intermediate | 1 |
|  |  |  | High | ≥2 |
| Seiler | Previous major bleeding (1); Active cancer (1); Hb <130 g/L in men or <120 g/L in women (1); Thrombocytopenia (<100×10^9^/L) (1); APT/NSAIDs (1) | Low physical activity;  Poor INR control | Low | 0-1 |
|  |  |  | Intermediate | 2-3 |
|  |  |  | High | >3 |
| IMPROVE | Active GI ulcer (4.5); Recent bleed (<3 months) (4); Thrombocytopenia (<50×10^9^/L) (4); Age ≥75 (3.5); Hepatic failure (INR >1.5) (2.5); Renal failure (2.5); ICU/CCU admission (2.5); Central venous catheter (2); Rheumatic disease (2); Current cancer (2); Male (1) | NA | Low | <7 |
|  |  |  | High | ≥7 |
| RIETE | Recent major bleeding (<15 days) (2); Creatinine >1.2 mg/dL (1.5); Anemia (Hb <130 g/L in men or <120 g/L in women) (1.5); Cancer (1); Pulmonary embolism (1); Age >75 (1) | NA | Low | 0 |
|  |  |  | Intermediate | 1-4 |
|  |  |  | High | >4 |

Abbreviations: SBP, Systolic blood pressure; AST, Aspartate transaminase; ALT, Alanine transaminase; ALP, Alkaline phosphatase; ULN, Upper limit of normal value; APT, Antiplatelet therapy; NSAIDs, Non-steroidal anti-inflammatory drugs; Hb, Hemoglobin; GI, Gastrointestinal; eGFR, Estimated glomerular filtration rate; INR, International normalized ratio; ICU, Intensive care unit; CCU, Coronary care unit; TTR, Time in the therapeutic range; NA, not applicable.

^a^ The criteria for inapplicable risk factors include rare incidence rates, unavailability, or challenges in monitoring.

**Table S3: Risk categories in both LSCB and non-LSCB groups**

| **Risk score** | **Risk categories (LSCB Group, n=363), %** | | | **Risk categories (Non-LSCB Group, n=1089), %** | | |
| --- | --- | --- | --- | --- | --- | --- |
|  | **Low** | **Intermediate** | **High** | **Low** | **Intermediate** | **High** |
| HAS-BLED | 25.1 | 37.5 | 37.2 | 43.4 | 35.5 | 21.0 |
| ORBIT | 19.3 | 24.8 | 55.9 | 14.1 | 46.0 | 39.9 |
| ATRIA | 47.9 | 9.1 | 47.4 | 47.9 | 4.6 | 47.5 |
| HEMORR2HAGES | 9.9 | 64.2 | 25.9 | 29.4 | 60.7 | 10.0 |
| Shireman | 17.1 | 71.3 | 12.4 | 28.7 | 66.8 | 4.6 |
| OBRI | 1.7 | 72.5 | 26.2 | 5.1 | 88.0 | 7.1 |
| ACCP | 0.8 | 5.8 | 93.1 | 2.9 | 13.6 | 83.5 |
| VTE-BLEED | 9.4 | NA | 90.6 | 25.7 | NA | 74.3 |
| Hokusai | 4.7 | 35.0 | 60.3 | 8.6 | 35.3 | 56.1 |
| Seiler | 5.5 | 74.7 | 19.8 | 10.3 | 79.2 | 10.6 |
| IMPROVE | 62.0 | NA | 38.0 | 82.3 | NA | 17.7 |
| RIETE | 1.9 | 82.9 | 15.2 | 7.9 | 85.1 | 7.1 |

Abbreviations: NA, not applicable.

**Table S4: Evaluation of the available rating scales based on stratification into high and low/intermediate risk of bleeding**^a^

| **Risk score** | **AUCs**  **(95% CI)** | **Sensitivity**  **(95% CI)** | **Specificity**  **(95% CI)** |
| --- | --- | --- | --- |
| HAS-BLED | 0.62 (0.58-0.65) | 37.1 (32.0-42.5) | 79.0 (76.4-81.4) |
| ORBIT | 0.61 (0.57-0.64) | 56.1 (50.6-61.4) | 60.1 (57.1-63.0) |
| ATRIA | 0.65 (0.61-0.68) | 69.8 (64.6-74.6) | 52.5 (49.5-55.5) |
| HEMORR2HAGES | 0.68 (0.65-0.71) | 25.9 (21.4-30.9) | 89.6 (87.6-91.4) |
| Shireman | 0.62 (0.58-0.65) | 12.5 (9.3-16.5) | 95.4 (94.0-96.5) |
| OBRI | 0.66 (0.63-0.70) | 26.2 (21.7-31.2) | 93.0 (91.3-94.4) |
| ACCP | 0.65 (0.62-0.67) | 93.2 (89.8-95.4) | 16.4 (14.3-18.8) |
| VTE-BLEED | 0.65 (0.61-0.68) | 90.7 (87.0-93.4) | 25.7 (23.1-28.4) |
| Hokusai | 0.52 (0.49-0.56) | 60.1 (54.7-65.3) | 43.8 (40.9-46.8) |
| Seiler | 0.59 (0.56-0.62) | 19.9 (15.9-24.7) | 89.5 (95.6-97.8) |
| IMPROVE | 0.64 (0.60-0.68) | 38.3 (33.2-43.7) | 86.4 (82.2-89.8) |
| RIETE | 0.66 (0.63-0.69) | 65.7 (60.4-70.7) | 59.7 (56.7-62.6) |

Abbreviations: CI, Confidence interval; AUC, Area under the ROC curve.

^a^ Patients were stratified as having a high or a low/intermediate risk of bleeding based on the bleeding risk score used. Calculated values were used to determine the sensitivity and specificity.

**Table S5: Multicollinearity diagnostics for twelve independent risk factors**

| **Variables** | **B** | **OR（95%CI）** | **VIF** | **P** |
| --- | --- | --- | --- | --- |
| Old age (>65 y) | 0.514 | 1.47 (1.02-2.24) | 1.033 | 0.013 |
| Hepatic impairment | 0.528 | 1.69 (1.09-2.62) | 1.031 | 0.017 |
| Renal dysfunction | 0.550 | 1.81 (1.29-2.55) | 1.063 | 0.001 |
| Single antiplatelet | 0.578 | 1.91 (1.29-2.83) | 1.267 | 0.005 |
| Dual antiplatelet | 0.812 | 2.22 (1.11-4.43) | 1.272 | 0.005 |
| Hypocalcemia | 0.822 | 2.30 1.67-3.17) | 1.078 | <0.001 |
| Cefoperazone/latamoxef exposure | 0.868 | 2.41 (1.68-3.46) | 1.021 | <0.001 |
| PT or APTT >1.2×ULN | 0.906 | 2.44 (1.67-3.57) | 1.081 | <0.001 |
| Thrombocytopenia (Pt <75×10⁹/L) | 0.946 | 2.66 (1.47-4.84) | 1.020 | 0.002 |
| Active gastrointestinal ulcer | 1.120 | 2.89 (1.52-5.52) | 1.018 | 0.001 |
| Anemia (Hb <90 g/L) | 1.244 | 3.32 (2.31-4.79) | 1.137 | <0.001 |
| Hypoproteinemia (albumin <30 g/L) | 1.893 | 6.16 (4.44-8.56) | 1.108 | <0.001 |

Abbreviations: PT, prothrombin time; APTT, activated partial thromboplastin time; ULN, Upper limit of normal value; Pt, platelet count; Hb, hemoglobin;


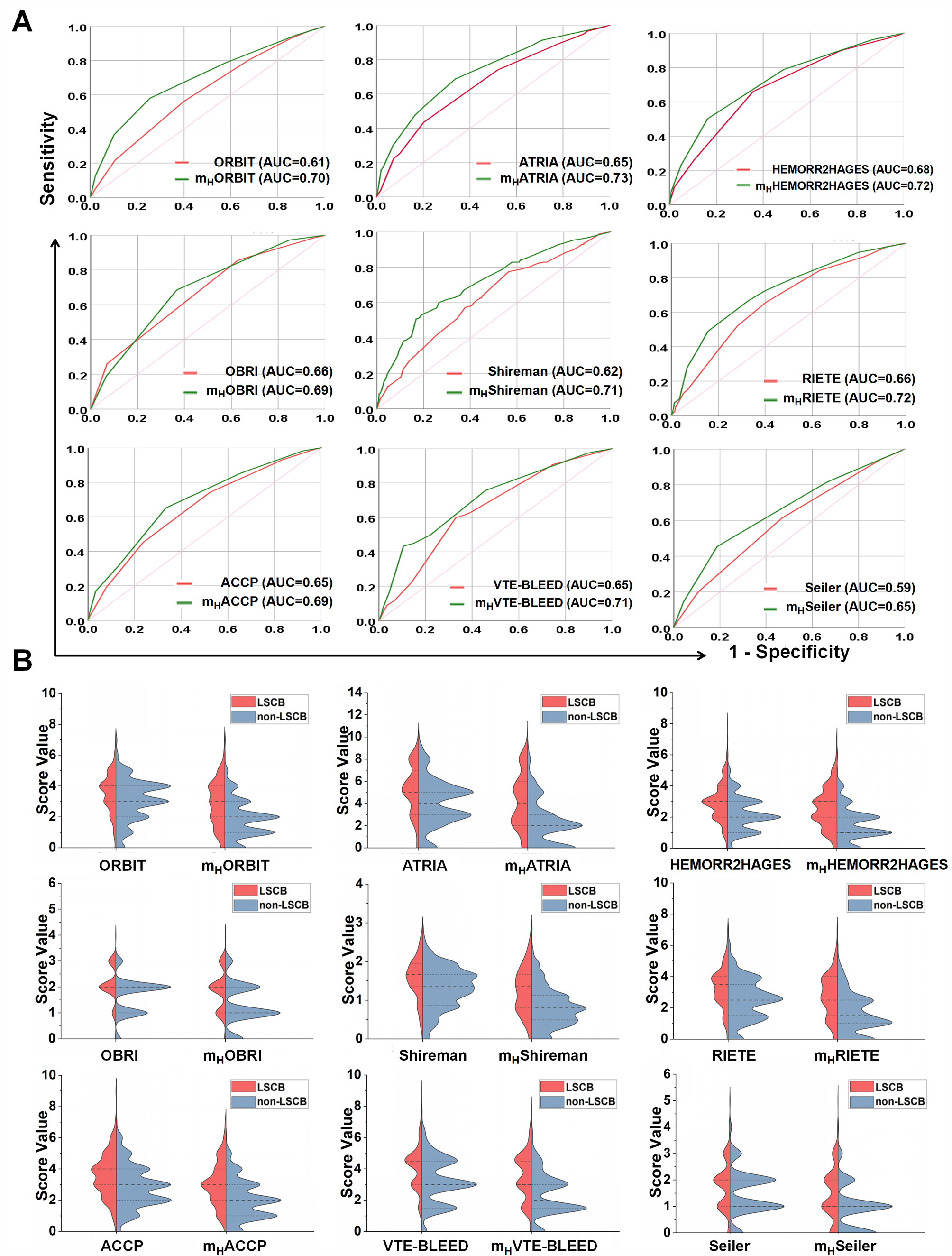


**Fig. S1: Impact of adjusted anemia inclusion criteria on the ROC curves.** (A) ROC curves before and after the adjustment of anemia inclusion criterion; (B) Alterations in score distribution following the adjustment of anemia inclusion criterion. m_H_ORBIT, m_H_ATRIA, m_H_HEMORR2HAGES, m_H_OBRI, m_H_Shireman, m_H_RIETE, m_H_ACCP, m_H_VTE-BLEED and m_H_Seiler: modified risk rating scales following the adjustment of anemia enrollment criterion.


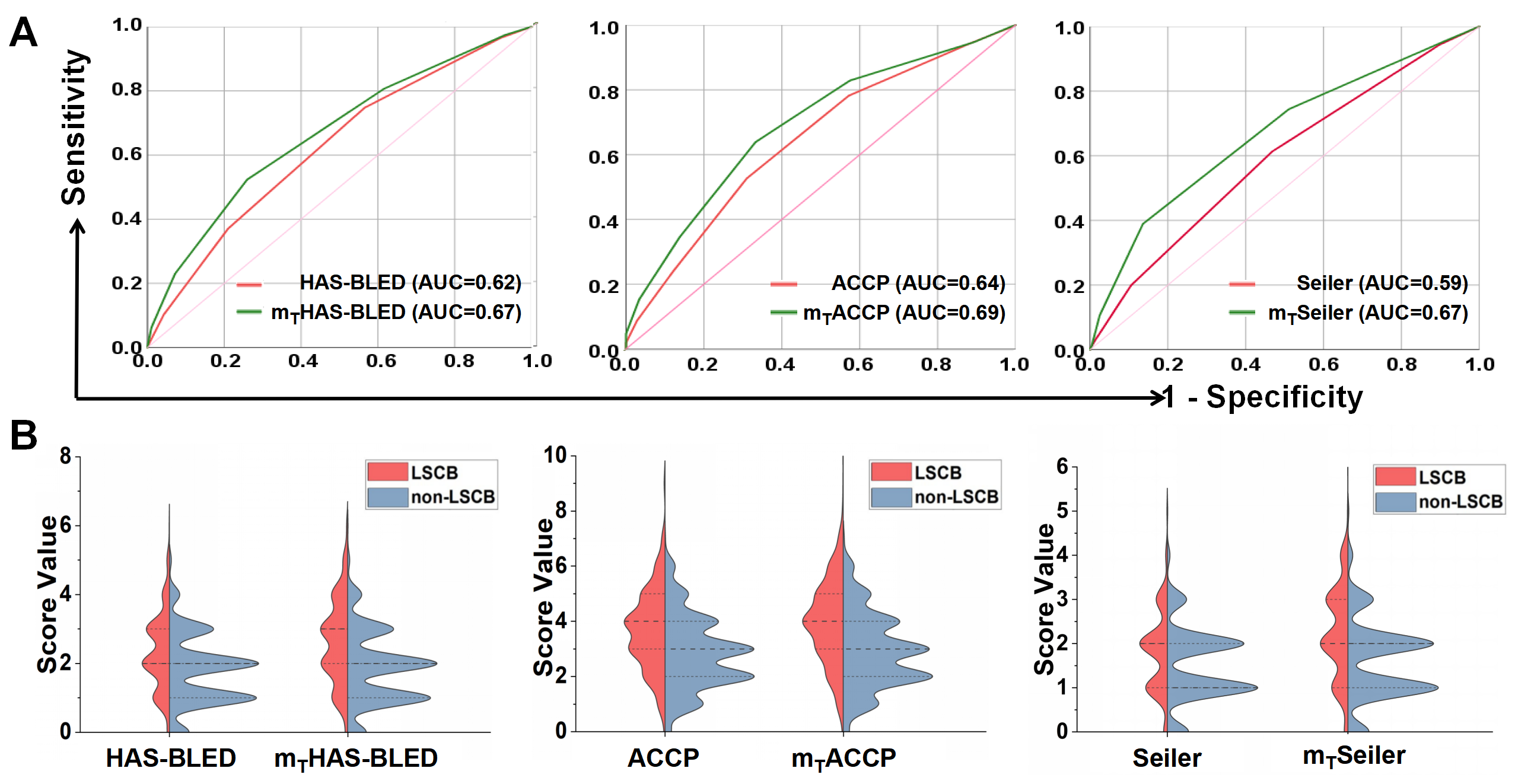


**Fig. S2: Impact of marker adjustment for coagulation disorders on ROC curves.** (A) ROC curves before and after the adjustment of the coagulation disorder inclusion criterion; (B) Alterations in score distribution following the adjustment of the coagulation disorder inclusion criterion. m_T_HAS-BLED, m_T_ACCP and m_T_Seiler: modified risk rating scales following the revision of coagulation disorder enrollment criterion.


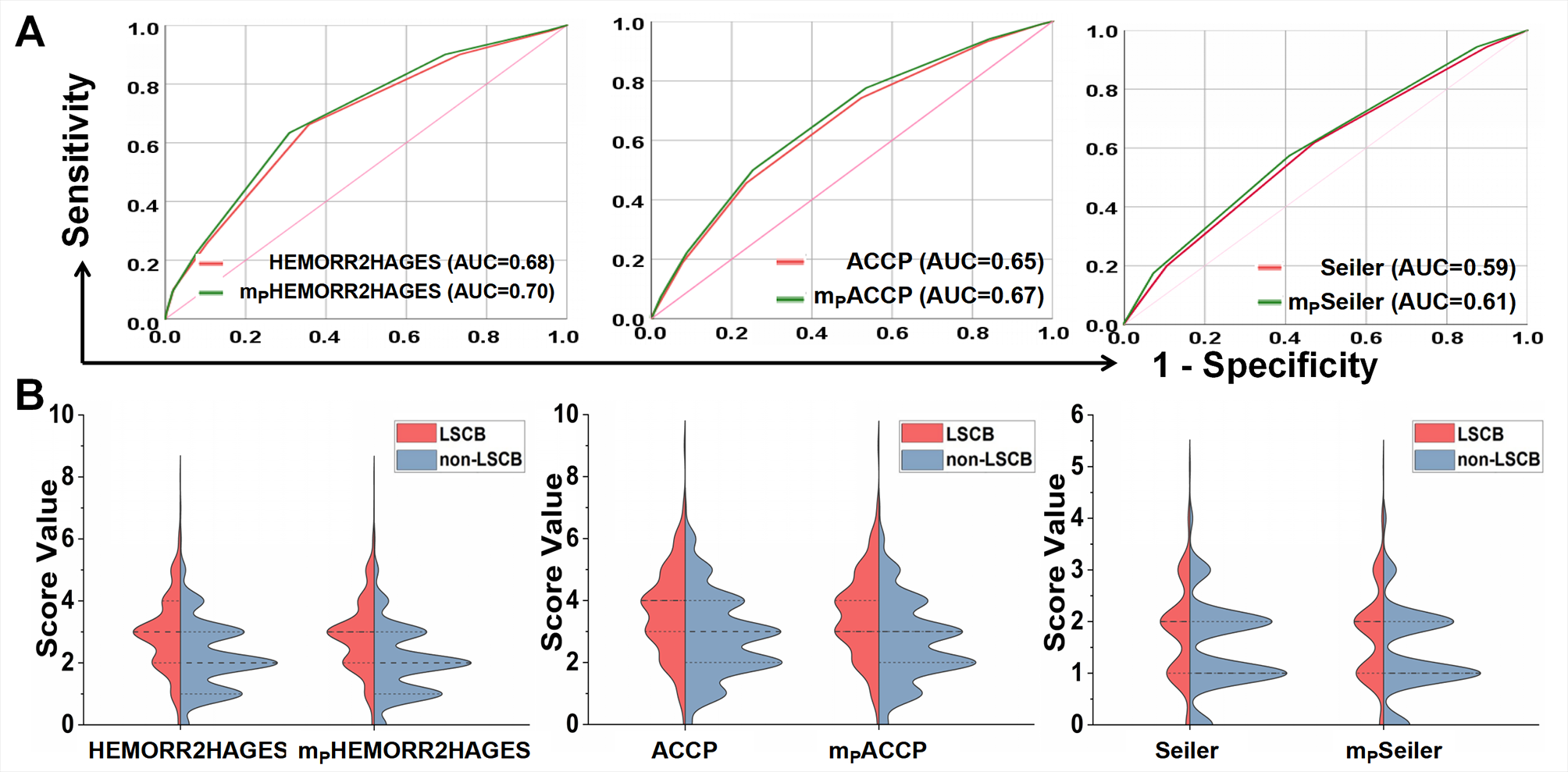


**Fig. S3: Impact of adjusted thrombocytopenia inclusion criteria on the ROC curves.** (A) ROC curves before and after the adjustment of thrombocytopenia inclusion criterion; (B) Alterations in score distribution following the adjustment of anemia inclusion criterion. m_P_HEMORR2HAGES, m_P_ACCP, m_P_Seiler: modified risk rating scales following the adjustment of thrombocytopenia enrollment criterion.


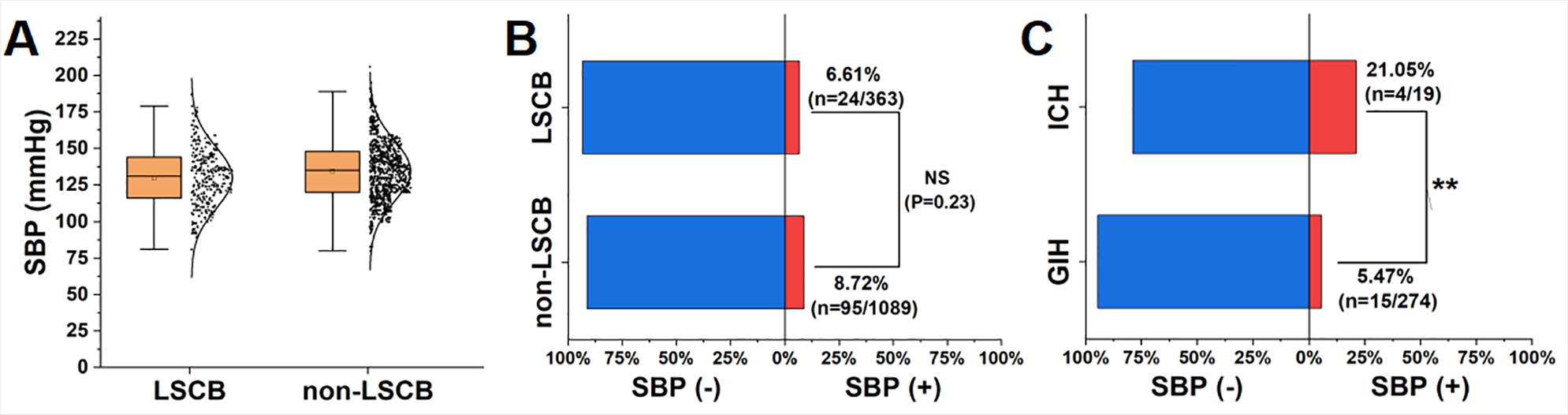


**Fig. S4: Effects of SBP >160mmHg on different types of LSCB events**. (A) Distribution of SBP in patients with and without LSCB; (B) Proportion of SBP >160mmHg in two cohorts; (C) Proportion of SBP >160mmHg in patients with ICH and GIH.

Abbreviations: SBP, systolic blood pressure; SBP (-), SBP ≤160mmHg before the LSCB events; SBP (+), SBP >160mmHg before the LSCB events; ICH, intracranial hemorrhage; GIH, gastrointestinal hemorrhage.
